# Supplementary material for: Triplex real-time PCR ZKIR-T assay for simultaneous detection of the Klebsiella pneumoniae species complex and identification of K. pneumoniae sensu stricto
Source: Microbiol Spectr. 2024 Oct 22;12(12):e00336-24. doi: 10.1128/spectrum.00336-24 (PMC11619374; doi:10.1128/spectrum.00336-24)
Supplement: Supplemental material — Tables S1 to S3. [file spectrum.00336-24-s0001.docx]

**Supplementary material**

**Table S1. Bacterial strains used to develop the triplex assay: 49 strains of the *K. pneumoniae* species complex (KpSc) representing phylogroups Kp1 to Kp7, and 19 *Klebsiella* strains that do not belong to the KpSC; all analyzed at INRAe.**

| Species (Phylogroup) | Strain name | Strain bank ID* | Sampling date | Source | Ct_zkir_P1 | Ct_zkir_P2 | Ct_Kp1_P |
| --- | --- | --- | --- | --- | --- | --- | --- |
| *Klebsiella pneumoniae* subsp*. pneumoniae* (Kp1) | SB4-2 | SB1067 | 2002 | Feces | **17.6** | U | **23.5** |
|  | ATCC13883^T^ | SB132 | n.a. | Blood | **16.4** | U | **22.7** |
|  | ATCC 700721 | SB107 | 1994 | Blood | **13.8** | U | **20.2** |
|  | none | SB1139 | 2002 | Feces | **15.1** | U | **20.9** |
|  | 5-2 | SB617 | 2000 | Natuurgebied canal | **15.6** | U | **21.3** |
|  | MIAE07651 | none | 2015 | Blood | **17.3** | U | **23.2** |
|  | 04A025 | SB20 | 1997 | Blood | **15.6** | U | **21.6** |
|  | 2-3 | SB612 | 2000 | Rijnhauwen bridge | **15.5** | U | **21.6** |
|  | BJ1-GA | SB4496 | 2011 | Liver abscess | **14.8** | U | **20.9** |
| *Klebsiella quasipneumoniae* subsp*. quasipneumoniae* (Kp2) | 01A030^T^ | SB11 | 1997 | Blood | **19.5** | U | U |
|  | none | SB1124 | 2002 | Canal water | **28.3** | U | U |
|  | U41 | SB2110 | 1990 | Environment | **26.7** | U | U |
|  | 10A442 | SB224 | 1998 | Blood | **18.5** | U | U |
|  | 99-1002 | SB2478 | 1999 | n.a. | **18.3** | U | U |
|  | 18A451 | SB255 | 1998 | Blood | **17** | U | U |
|  | 11128 | SB3445 | n.a. | Diarrhoea | **20** | U | U |
|  | CRBIP28.152 (18A69) | SB59 | 1997 | Blood | **26.1** | U | U |
|  | KlebAli 0320584 | SB98 | n.a. | Environment | **18.8** | U | U |
| *Klebsiella variicola* subsp. *variicola* (Kp3) | 01A065 | SB1 | 1997 | Blood | **22.9** | U | U |
|  | 07A058 | SB31 | 1997 | Blood | **21.6** | U | U |
|  | IPEUC-1516 | SB3278 | 1988 | Urine | **22.9** | U | U |
|  | CIP 53.24 | SB3295 | n.a. | n.a. | **22.5** | U | U |
|  | CIP 53.26 (1756/51) | SB3301 | n.a. | n.a. | **23.3** | U | U |
|  | F2R9^T^ | SB48 | n.a. | Banana | **22.7** | U | U |
|  | 6115 (KLSP49) | SB489 | n.a. | n.a. | **21.7** | U | U |
|  | 4425/51 | SB497 | n.a. | n.a. | **21.8** | U | U |
|  | Kp342 | SB579 | n.a. | Maize | **21.7** | U | U |
| *Klebsiella quasipneumoniae* subsp. *similipneumoniae* (Kp4) | CRBIP28.12 (09A323) | SB164 | 1997 | Blood | **26** | U | U |
|  | 12A476 | SB203 | 1998 | Blood | **25.5** | U | U |
|  | 07A044^T^ | SB30 | 1997 | Blood | **25.6** | U | U |
|  | 325 | SB3233 | 1975 | n.a. | **23.7** | U | U |
|  | CIP 52.200 (1303/50) | SB3297 | n.a. | n.a. | **23.9** | U | U |
|  | 1303/50 (KLSP50) | SB490 | n.a. | n.a. | **25.1** | U | U |
|  | 4463/52 (KLSP60) | SB500 | n.a. | n.a. | **24.2** | U | U |
|  | CIP110288 | SB4697 | 2010 | Farmland soil | **26.6** | U | U |
|  | 1-1 | SB610 | 2000 | Lake kikker | **25.6** | U | U |
| *Klebsiella variicola* subsp*. tropica* (Kp5) | CDC 4241-71 | SB94 | n.a. | Environment | **22.4** | U | U |
|  | Gal12 | SB824 | n.a. | Environment | **22.3** | U | U |
|  | 814 | SB5387 | 2015 | Fecal sample | **20.3** | U | U |
|  | 885 | SB5439 | 2016 | n.a. | **25.9** | U | U |
|  | 1266^T^ | SB5531 | 2016 | Fecal sample | **29.9** | U | U |
|  | 1283 | SB5544 | 2016 | Fecal sample | **29.7** | U | U |
|  | 1375 | SB5610 | 2016 | Fecal sample | **19.2** | U | U |
| *Klebsiella quasivariicola* (Kp6) | 08A119 | SB33 | 1997 | Blood | U | **27** | U |
|  | 10982 | SB6071 | 2005 | Peri-rectal | U | **26.3** | U |
|  | 01-467-2ECBU | SB6094 | 2015 | Feces | U | **26.1** | U |
|  | 01-310A | SB6095 | 2013 | Vaginal swab | U | **27** | U |
|  | KPN1705^T^ | SB6096 | 2014 | Wound | U | **26.2** | U |
| *Klebsiella africana* (Kp7) | 200023^T^ | SB5857 | 2016 | n.a. | **17.3** | U | U |
| *Klebsiella michiganensis* (Ko1) | CIP 110787^T^ | SB4934 | 2010 | n.a. | U | U | U |
|  | 05A071 | SB71 | 1997 | n.a. | U | U | U |
|  | 09A029 | SB78 | 1997 | n.a. | U | U | U |
| *Klebsiella grimontii* (Ko6) | 07A479 | SB324 | 1998 | n.a. | U | U | U |
|  | 06D090 | SB352 | 1998 | n.a. | U | U | U |
|  | 06D021^T^ | SB73 | 1997 | n.a. | U | U | U |
| *Klebsiella oxytoca* (Ko2) | ATCC 13182^T^ | SB175 | n.a. | n.a. | U | U | U |
|  | 02A067 | SB131 | 1997 | n.a. | U | U | U |
|  | NCTC 49131 | SB136 | n.a. | n.a. | U | U | U |
| *Klebsiella terrigena^R^* | ATCC33257^T^ | SB170 | n.a. | n.a. | U | U | U |
|  | 17C143 | SB313 | 1998 | n.a. | U | U | U |
|  | V9813596 | SB2796 | 1998 | n.a. | U | U | U |
| *Klebsiella planticola^R^* | 01A041 | SB7 | 1997 | n.a. | U | U | U |
|  | ATCC33531^T^ | SB174 | n.a. | n.a. | U | U | U |
|  | 12C169 | SB303 | 1998 | n.a. | U | U | U |
| *Klebsiella ornithinolytica^R^* | ATCC31898^T^ | SB171 | n.a. | n.a. | U | U | U |
| *Klebsiella aerogenes* | CIP 60.86T | SB3629 | n.a. | n.a. | U | U | U |
|  | 01A089 | SB538 | 1997 | n.a. | U | U | U |
|  | 02A002 | SB539 | 1997 | n.a. | U | U | U |

U stands for undetermined, i.e. no amplification; Ct: cycle threshold; T indicates a type strain; n.a: not available; R: also called *Raoultella* spp. according to Drancourt et al. 2001. Int J Syst Evol Microbiol 51:925–932.

**Table S2. Control strains representing each of the seven phylogroups from *K. pneumoniae* species complex used at SSI for the triplex PCR**

| Strain name | Original Strain name | Phylogroup | Species |
| --- | --- | --- | --- |
| MVK-06H079 | ATCC 13883^T^ | Kp1 | *K. pneumoniae* |
| MVK-06H171 | ATCC 700721 (MGH 78578) | Kp1 | *K. pneumoniae* |
| MVK-06H172 | BIP28.152 | Kp2 | *K. quasipneumoniae* subsp*. quasipneumoniae* |
| MVK-06H173 | CIP 53.26 (1756/51) | Kp3 | *K. variicola* subsp*. variicola* |
| MVK-06H174 | CIP 52.200 (1303/50) | Kp4 | *K. quasipneumoniae* subsp*. similipneumoniae* |
| MVK-06H175 | CDC 4241-71 | Kp5 | *K. variicola* subsp. *tropica* |
| MVK-06H176 | 08A119 | Kp6 | *K. quasivariicola* |
| MVK-06H177 | 200023^T^ | Kp7 | *K. africana* |

^T^ indicates a type strain.

**Table S3. Characterization and Ct values of 46 test strains representing phylogroups Kp1 to Kp6, and eight control strains, analyzed at SSI. Rep1 and Rep2 are two technical replicates of the same sample. Rep3 is a biological replicate**.

| Strain name | Sampling date | Source | ST | Species (PhG) ^1^ | Ct_zkir_P1 | | | Ct_zkir_P2 | | | Ct_Kp1_P | | |
| --- | --- | --- | --- | --- | --- | --- | --- | --- | --- | --- | --- | --- | --- |
|  |  |  |  |  | **Rep1** | **Rep2** | **Rep3** | **Rep1** | **Rep2** | **Rep3** | **Rep1** | **Rep2** | **Rep3** |
| MVK-06S001 | feb-18 | Sewage | ST391 | *K. pneumoniae* (Kp1) | **18.2** | **17.8** | **18.1** | U | U | U | **20.9** | **21** | **21.3** |
| MVK-06S005 | feb-18 | Sewage | ST391 |  | **18.1** | **18.1** | **18** | U | U | U | **21.1** | **21.7** | **21.5** |
| MVK-06S008 | feb-18 | Sewage | ST234 |  | **17.6** | **17.8** | **18** | U | U | U | **20.6** | **21.4** | **21.4** |
| MVK-06S009 | feb-18 | Sewage | ST976 |  | **17.2** | **17.6** | **18.1** | U | U | U | **20.3** | **21.2** | **21.5** |
| MVK-06S010 | feb-18 | Sewage | ST5765 |  | **17.5** | **17.4** | **18.4** | U | U | U | **20.5** | **20.9** | **21.9** |
| MVK-06S013 | feb-18 | Sewage | ST252 |  | **17.6** | **17.6** | **18.5** | U | U | U | **20.6** | **20.9** | **21.6** |
| MVK-06G003 | jun-18 | Animal carriage | ST5 |  | **17.6** | **18.1** | **18.5** | U | U | U | **20.6** | **21.4** | **21.6** |
| MVK-06G005 | jun-18 | Animal carriage | ST5 |  | **17.3** | **17.8** | **18.1** | U | U | U | **20.2** | **21.3** | **21.3** |
| MVK-06G009 | jun-18 | Animal carriage | ST661 |  | **17.8** | **17.5** | **18.3** | U | U | U | **20.9** | **20.9** | **21.8** |
| MVK-06G014 | jun-18 | Animal carriage | ST661 |  | **17.7** | **17.6** | **18.3** | U | U | U | **20.8** | **21.1** | **21.7** |
| MVK-06G017 | jun-18 | Animal carriage | ST46 |  | **17.8** | **17.8** | **18.6** | U | U | U | **20.5** | **20.6** | **21.6** |
| MVK-06G018 | jun-18 | Animal carriage | ST5 |  | **17.8** | **17.6** | **18.4** | U | U | U | **20.5** | **20.9** | **21.5** |
| MVK-06G021 | jun-18 | Animal carriage | ST661 |  | **17.7** | **17.7** | **18.8** | U | U | U | **20.7** | **21** | **22.2** |
| MVK-06G024 | jun-18 | Animal carriage | ST661-1LV^1^ |  | **17.9** | **18** | **18.4** | U | U | U | **21.1** | **21.5** | **21.8** |
| MVK-06S016 | sep-18 | Sewage | ST391 |  | **17.6** | **17.1** | **18.2** | U | U | U | **21.4** | **21.1** | **21.5** |
| MVK-06S023 | sep-18 | Sewage | ST45 |  | **18.4** | **17.4** | **18.2** | U | U | U | **21.7** | **20.9** | **21.5** |
| MVK-06S029 | sep-18 | Sewage | ST391 |  | **17.6** | **17.6** | **18.1** | U | U | U | **20.5** | **21** | **21.4** |
| MVK-06S033 | sep-18 | Sewage | ST889 |  | **17.5** | **17.8** | **18.1** | U | U | U | **20.6** | **21.2** | **21.6** |
| MVK-06S038 | sep-18 | Sewage | ST273-2LV^2^ |  | **17.4** | **17.5** | **18** | U | U | U | **20.7** | **21.3** | **21.4** |
| MVK-06S049 | sep-18 | Sewage | ST20-1LV^1^ |  | **17.4** | **17.8** | **17.9** | U | U | U | **20.8** | **21.7** | **21.2** |
| MVK-06S045 | sep-18 | Sewage | ST4756 | *K. quasipneumoniae* subsp. *quasipneumoniae* (Kp2) | **28.6** | **27.7** | **29.2** | U | U | U | U | U | U |
| MVK-06S012 | feb-18 | Sewage | ST5766 | *K. variicola* subsp. *variicola* (Kp3) | **25.3** | **24.5** | **25.5** | U | U | U | U | U | U |
| MVK-06S024 | sep-18 | Sewage | ST5065 |  | **25.1** | **24.4** | **26.5** | U | U | U | U | U | U |
| MVK-06S035 | sep-18 | Sewage | ST285 |  | **U** | **37.2** | **U** | U | U | U | U | U | U |
| MVK-06S039 | sep-18 | Sewage | ST6110 |  | **24.5** | **23.5** | **24.6** | U | U | U | U | U | 35.2 |
| MVK-06H010 | apr-18 | Human clinical (feces) | ST3982 |  | **25.8** | **25.4** | **26.3** | U | U | U | U | U | U |
| MVK-06H054 | may-18 | Human clinical (feces) | ST641 |  | **25.8** | **25.2** | **26.4** | U | U | U | U | U | U |
| MVK-06H070 | may-18 | Human clinical (feces) | ST1562 |  | **24.9** | **24.8** | **26.4** | U | U | U | U | U | U |
| MVK-06H077 | jun-18 | Human clinical (feces) | ST1915 | *K. variicola* subsp. *variicola* (Kp3) | **25.3** | **24.7** | **25.8** | U | U | U | U | U | U |
| MVK-06F135 | mar-19 | Food (salad) | ST146 |  | **25** | **25.1** | **25.6** | U | U | U | U | U | U |
| MVK-06F137 | mar-19 | Food (salad) | ST363 |  | **25** | **31.4** | **25.6** | U | U | U | U | U | U |
| MVK-06F149 | mar-19 | Food (salad) | ST662 |  | **24.9** | **24.2** | **26.4** | U | U | U | U | U | U |
| MVK-06H098 | may-19 | Human clinical (blood) | ST5771 |  | **24.1** | **23.8** | **26.1** | U | U | U | U | U | U |
| MVK-06H100 | may-19 | Human clinical (blood) | ST250 |  | **24.5** | **23.8** | **25.9** | U | U | U | U | U | U |
| MVK-06H113 | may-19 | Human clinical (blood) | ST355 |  | **22.9** | **22** | **26.2** | U | U | U | U | U | U |
| MVK-06H116 | may-19 | Human clinical (blood) | ST2594 |  | **24.8** | **23.5** | **26.5** | U | U | U | U | U | U |
| MVK-06H127 | may-19 | Human clinical (blood) | ST355 |  | **24** | **23.5** | **25.8** | U | U | U | U | U | U |
| MVK-06H156 | may-19 | Human clinical (blood) | ST5778 |  | **24.2** | **23.9** | **25.9** | U | U | U | U | U | U |
| MVK-06H166 | may-19 | Human clinical (blood) | ST5779 |  | **24.4** | **24.1** | **25.7** | U | U | U | U | U | U |
| MVK-06H167 | may-19 | Human clinical (blood) | ST208 |  | **25.1** | **24.5** | **26.5** | U | U | U | U | U | U |
| MVK-06H168 | may-19 | Human clinical (blood) | ST5798 |  | **33.7** | **32.2** | **34.5** | U | U | U | U | 37.8 | U |
| MVK-06F021 | sep-18 | Food (meat) | ST3450 | *K. quasipneumoniae* subsp. *similipneumoniae* (Kp4) | **36.1** | **35.3** | **37.4** | U | U | U | U | U | U |
| MVK-06S018 | sep-18 | Sewage | ST367 |  | **36.4** | **33** | **37** | U | U | U | U | U | U |
| MVK-06H092 | may-19 | Human clinical (blood) | ST5770 |  | **39.7** | **37.4** | **36.3** | U | U | U | U | U | U |
| MVK-06H095 | may-19 | Human clinical (blood) | ST1822 |  | **35** | **32.3** | **36.2** | U | U | U | U | U | U |
| MVK-06H101 | may-19 | Human clinical (blood) | ST4973 | *K. quasivariicola* (Kp6) | U | U | U | **26.8** | **26.7** | **27.7** | U | U | U |
| MVK-06H171 |  | Control strain |  | *K. pneumoniae* (Kp1) | **20.2** | **20.2** | **18.3** | U | U | U | **24** | **24** | **21.5** |
| MVK-06H079 |  | Control strain |  | *K. pneumoniae* (Kp1) | **18.3** | **17.9** | **18.4** | U | U | U | **21** | **21.2** | **21** |
| MVK-06H172 |  | Control strain |  | *K. quasipneumoniae* subsp. *quasipneumoniae* (Kp2) | **29.1** | **28.2** | **28.7** | U | U | U | U | U | U |
| MVK-06H173 |  | Control strain |  | *K. variicola* subsp. *variicola* (Kp3) | **24.5** | **24** | **26** | U | U | U | U | U | U |
| MVK-06H174 |  | Control strain |  | *K. quasipneumoniae* subsp. *similipneumoniae* (Kp4) | **32.6** | **31.4** | **35.1** | U | U | U | U | U | U |
| MVK-06H175 |  | Control strain |  | *K. variicola* subsp. *tropica* (Kp5) | **24.2** | **24** | **25.6** | U | U | U | U | U | U |
| MVK-06H176 |  | Control strain |  | *K. quasivariicola* (Kp6) | U | U | U | **27.3** | **27.4** | **28.2** | U | U | U |
| MVK-06H177 |  | Control strain |  | *K. africana* (Kp7) | **21.1** | **21.1** | **22.5** | U | U | U | U | U | U |

Positive control strains representing all phylogroups are numbered sequentially from MVK-06H171 to MVK-06H177.

Rep1-3 denote replicates, U stands for Undetermined, i.e. no amplification; ST: Sequence Type; Ct: cycle threshold

The unexpected results are marked with red font and are addressed in the text.

^1^partial *phoE*; ^2^ partial *phoE* and *mdh;* the ST may correspond to the one indicated or to a single locus variant
